# Enhanced repeated measurement of psychological tasks and form questions via a web-based mobile app

**DOI:** 10.1539/eohp.2025-0019

**Published:** 2025-10-09

**Authors:** Yuki Nishimura, Hiroki Ikeda, Shun Matsumoto, Shuhei Izawa, Xinxin Liu, Atsuko Tamaoki, Tomohide Kubo

**Affiliations:** 1 Research Center for Overwork Related Diseases, National Institute of Occupational Safety and Health, Kawasaki, Japan

**Keywords:** fatigue management, mobile application, psychological tests, remote surveys

## Abstract

Traditional paper-and-pencil methods have limitations for data collection in occupational health research. Here, we introduce a new web-based version (Fatigue checker web-ver.) of an application designed to better facilitate survey management and communication with participants. The application operates on mobile web browsers and integrates with LINE, Japan’s most popular messaging app, and contains customizable forms, psychological tasks such as the Psychomotor Vigilance Task (PVT), Flanker Task, and n-back Task. Daily schedule acquisition is achieved via a dedicated user interface. Cross-correlation analysis showed fair synchronization between the app and gold-standard PVT equipment, indicating the app’s reliability in measuring reaction times. Specifically, the analysis revealed significant cross-correlation for mean reaction time, median reaction time, mean reciprocal reaction time, and number of lapses, which indicates that the app can reliably measure fatigue and alertness levels. Since its release, the app has been successfully employed collecting high-quality data in six projects involving 571 participants. Despite minor issues, the app significantly improves the efficiency and accuracy of remote surveys, offering valuable insights into workers’ health and safety. It also enables occupational health and safety specialists to monitor workers and workplaces from a broader perspective than traditional methods. The public version of the app also enables every worker to monitor their own occupational health and safety status. Insights from repeated self-checking of fatigue will promote better working conditions and environments.

## Background and issue

Occupational fatigue is a critical issue for safety and health at work. Due to societal changes, fatigue is now a long-term rather than short-term problem. Thus, better methods are required to study the long-term effects of working conditions on fatigue among modern workers. Collecting reliable and comprehensive data from actual workers is challenging for occupational health professionals and researchers. Conducting multiple measurements and acquiring behavioral data are critical but require considerable effort from both the administrative and participant sides.

The traditional paper-and-pencil method has been employed for this purpose for decades but has multiple drawbacks in terms of its physical limitations. Although this method may have a lower burden to participate than digitized modern methods, papers can quickly become disorganized or damaged, and there are issues regarding logistical and spatial inconvenience. Difficulty in maintaining anonymity is one of the other issues. Regarding data quality, the paper-and-pencil method also suffers from missing and incorrect answers due to the lack of an automatic checking function. Problems originating from physical aspects also arise when collecting behavioral data from psychological tests.

In contrast, the rapid spread of smartphones has enabled us to obtain frequent, repeated, and accurate measurements, even among busy workers. The introduction of online form services, such as Google Forms and SurveyMonkey, effectively addressed most of the problems of the paper-and-pencil method, particularly for surveys targeting remote participants. Although some participants who are less familiar with modern digital devices may find it challenging to participate^1)^, online surveys remain the preferred method for conducting broad, accurate, and convenient surveys^2)^. Online forms still have some limitations, particularly in conducting repeated measures and accurate behavioral tasks. Manual input of participant ID by participants is the typical method for multiple measurements, but participants may forget or incorrectly input their ID, resulting in missing or unreliable data.

Questionnaires are a valuable tool for gathering self-reported data, but are limited by response biases, such as recall bias and the subjective nature of self-reflection. Behavioral data collected through psychological tasks offer several advantages: objectivity, deeper insights, and increased engagement^3)^. As behavioral data require an extremely accurate recording of participant reactions, a dedicated measurement device is often the best choice, but not always the first. Following the rapid development of personal computers, the use of specialized software, such as E-prime (Psychology Software Tools, Inc., Sharpsburg, PA, USA) or Presentation (Neurobehavioral Systems, Inc., Albany, CA, USA), running on personal computers has become the primary choice for conducting psychological tasks. Multiple open-source packages, such as Python-based PsychoPy (Open Science Tools Ltd., Nottingham, England) and MATLAB-based Psychtoolbox (Psychtoolbox@github), have also been developed for accurate stimulus presentation and reaction recording.

Since the late 90s, web-based psychological experiments have become increasingly popular^4)^. Several web-based frameworks are currently available for conducting psychological studies, including PsychoJS, jsPsych, and Lab.js, among others^5)^. Although their precision and lag metrics are slightly worse than those of lab-based options, web-based experiments have become more popular because they have advantages in terms of providing access to a broader population, lower costs, and absence of time limitations^4)^. The frameworks are developed to work primarily with PC web browsers; however, they can run on mobile devices with slight modifications if the mobile browser supports JavaScript.

In the mid-2010s, a research team at the National Institute of Occupational Safety and Health, Japan, developed an Android application to administer the PVT and collect study data from fatigue questionnaires, daily schedules, and the Visual Analogue Scale^6)^. The integration of this native application has significantly enhanced the research process. However, due to the high adoption rate of iOS in Japan (around 60%) and the absence of the app on Google Play, it was necessary to distribute Android tablets to participants with the application pre-installed. Moreover, there was limited flexibility in modifying the questionnaire items.

## Viewpoints on improvement

To address these issues, we developed “Fatigue checker web-ver.”, which runs on mobile web browsers. As most workers own a smartphone and have sufficient internet access, the enormous burden of conducting large and remote surveys is alleviated by the use of electronic means.

To ensure the reliability of psychological tasks, we selected the jsPsych package^7)^ for conducting these tasks on participant-owned devices. The package is widely adopted in academia and has undergone extensive accuracy validation^5)^.

A large-scale remote survey also requires managing various inquiries from participants, not only during the survey period but also before and after it. In order to simplify communication between researchers and participants, the new app can be linked to LINE (LY Corporation, Tokyo, Japan), the most popular messaging app in Japan, through its Application Programming Interface (API). Integration with the LINE app enables researchers to communicate with participants, send notifications for periodic measurements, and display personalized interfaces that provide convenient access to features, such as survey windows and portal pages directly to participants’ devices.

Thus, development of the new web-based survey application alleviates the significant burdens that existed in the past. It is capable of participant management, can be integrated with a popular messaging app, and has shown high reliability in task measurements, as these are run by jsPsych.

## Implementation

**Fig. 1.**
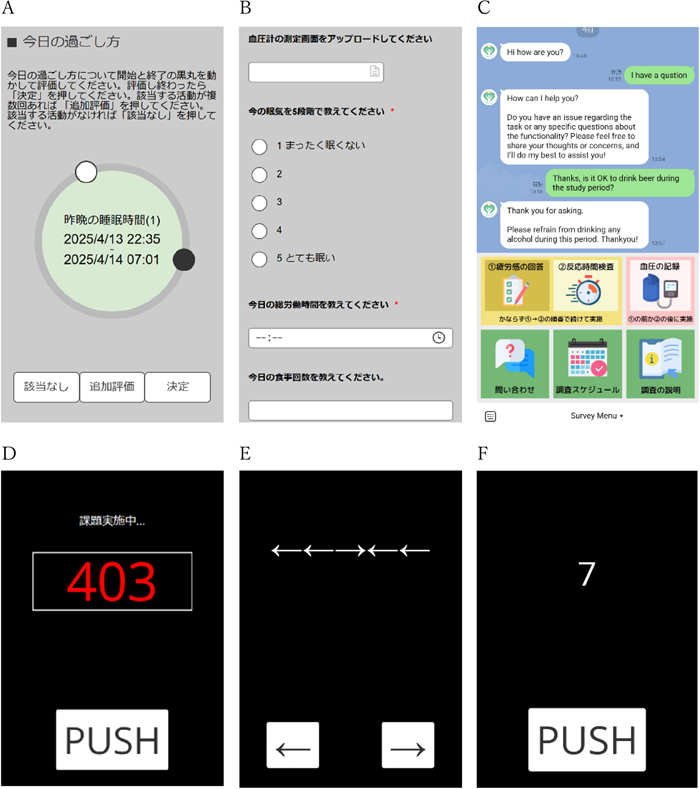
Examples of the survey interface **A**: User interface for entering the daily time schedule. **B**: Form survey window showing file upload, radio button, time format, and simple number format questions. **C**: User window of the LINE communication app showing quick-link navigation buttons (rich-menu) in the lower half of the screen. **D**: Psychomotor Vigilance Task window. Participants respond by tapping the PUSH button as quickly as possible when the red counter appears on the screen. **E**: Flanker task window. Participants respond by tapping either of the arrow buttons corresponding to the direction of the arrow displayed in the center (target stimuli) as quickly as possible while ignoring the other arrows (distractors). **F**: n-back task window. Participants respond by pressing the PUSH button as quickly as possible when the displayed number matches the number presented n trials ago.

The app can acquire data from the following options: 1) Online forms (similar to primary online form services, such as Google Forms and Microsoft Forms) for questionnaires, 2) Built-in psychological tasks (PVT, Flanker Task, and n-back Task) for behavioral data, and 3) A dedicated user interface for acquiring the daily schedule. To prevent data loss, all measured data are sent to the server as soon as the measurement ends. Figure 1 shows examples of the user interface of the above options.

To ensure that behavioral results are reported accurately, all psychological tasks are run by the jsPsych package^7)^. Of the built-in tasks, the PVT is widely used to objectively measure alertness level (vigilance) by asking the participant to press a button as soon as the simple digital counter starts ticking^8)^. Parameters such as task length and lapse threshold can be customized to conduct a 10-, 5-, or 3-minute (or any desired length) version of the PVT. The Flanker task measures the effect of distracting information on cognitive processing, assessing attentional control by requiring participants to focus on a target stimulus while ignoring adjacent distractors^9)^. The n-back task assesses cognitive control and memory capacity, and measures working memory by having participants identify when the current stimulus matches one from “n” steps earlier^10)^. The three tasks included in the app for measuring fatigue have been employed for assessing vigilance, attention, and executive functions in numerous other research fields. There is provision for additional tasks written in JavaScript to be added to the study’s task battery.

There are two pathways to participate in a survey. First, anyone can self-register as a participant if they know the project URL issued for the particular study project. The study manager (researcher) can view and manage all self-registered participants using the Participant Management Console. Second, the console can be used to pre-register participants and generate a unique participation URL for each participant. Thus, the manager can pre-register all participants beforehand and distribute the URL, which directly navigates to the survey menu as a text link or QR code^a^, thereby reducing possible hassles related to self-registration.

LINE is Japan’s most popular messaging mobile application, and offers a web API for interacting with its users. After a participant with an active LINE account has registered the “Official Account”^11)^ of the study project as their “friend” in their LINE app, the web application can be used as an interface for communication through LINE chat, and a “rich-menu” (eg, buttons with custom hyperlinks) is presented in their chat window (Figure 1C).

The web app is hosted on Firebase, Google’s mobile and web app development platform, taking advantage of its low costs, high security, and high service stability. Materials are distributed as webpages with JavaScript codes to run tasks, allowing recent Android and iOS devices to conduct measurements through a mobile web browser. No additional installation is required. One measurement consumes only a few hundred KB to less than 1 MB of capacity (net transferred data without cache). All data are securely stored in encrypted Firebase storage until the end of the survey period. In addition, all communication with the server is encrypted using a TLS connection.

aQR Code is a registered trademark of DENSO WAVE INCORPORATED, Aichi, Japan.

## Effect, impact, and outcomes

Six projects have been completed since the release of the application, targeting a total of 571 participants. The total data collected using the application reached 24,675 records (14,377 for Forms and 10,298 for psychological tasks, such as PVT) as of April 2025, including previously published work^12)^. Conducting a survey of this scale is not possible without the features of the new app.

Prompt data uploads throughout the survey period of each measurement of every participant enabled researchers to observe participants’ adherence to survey requirements such as timing, the number of measurements, and task completion. This monitoring capability allowed early detection and intervention to reduce missing data. All projects were completed with the anticipated quality of data.

A comparison of data measured by the app with data acquired using gold-standard PVT equipment (PVT-192; Ambulatory Monitoring Inc., Ardsley, NY, USA) in a laboratory setting confirmed high synchronization. We analyzed data from hourly PVT measurements alternately using the app and the PVT-192. Except for one participant who dropped out during the experiment, 12 participants performed repeated PVT measurements using the app (3-min brief version PVT, also known as PVT-B^13)^) and the PVT-192 (10-min original version^14)^) during a consecutive 40-hour continuous sleep deprivation experiment. Measurement started with the app and transitioned to PVT-192 after 1 hour, then continued to alternate every hour until the end of the experiment (except during meal breaks). The research ethics review committee of the National Institute of Occupational Safety and Health, Japan, approved all procedures (2023N02). Cross-correlation analysis of the fitted value of the linear mixed model, using 15 and 14 time points for the app and the PVT-192, respectively, showed the most significant cross-correlation factors (CCF) at lag = 0.5 (CCF>0.85 for all indices; as no measurements were conducted using both devices simultaneously, CCF at lag = 0 cannot be calculated). A CCF greater than 0.8 is generally interpreted as indicating a very high degree of temporal synchrony. The more positive the lag value, the more it indicates that the app’s data preceded the PVT-192’s data. The current results indicate that data from the app and PVT-192 were synchronized with the minimum possible lag in the current design. Table 1 lists the results of cross-correlation analysis for mean reaction time (RT), median RT, mean reciprocal RT (RRT; 1/sec), and number of lapses (RT >355 ms and RT >500 ms for the 3-min app version and the 10-min PVT-192, respectively). The CCF ranged from -1 to 1, similar to the ordinal correlation factor. High CCF values at lag = 0 and low (near 0) values at other lags indicate synchronization in vigilance measured by the app’s PVT and PVT-192 across all indices, at least in a laboratory setting. The time series of each index are listed in eFigure 1.

**Table 1.** Results of cross-correlation analysis between PVT indices measured by the app’s PVT and the PVT-192

	Cross-correlation factor							
	Lag −3.5	Lag −2.5	Lag −1.5	Lag −0.5	Lag 0.5	Lag 1.5	Lag 2.5	Lag 3.5
meanRT (ms)	−0.20	0.12	0.55*	0.63*	0.87*	0.65*	0.33	0.09
medianRT (ms)	−0.28	−0.10	0.00	0.42	0.99*	0.48	0.10	−0.12
meanRRT (1/sec)	−0.22	0.10	0.43	0.65*	0.92*	0.80*	0.56*	0.29
lapse (times)	−0.33	−0.04	0.21	0.55*	0.87*	0.85*	0.74*	0.48

PVT, Psychomotor Vigilance Task; RT, reaction time; RRT, reciprocal reaction time.

N=12 (320 valid observations).

* Significant cross-correlation *p*<0.05.

1 Lag = 1 hour. As the measurements were conducted using the app’s PVT function and PVT-192 alternately, cross-correlation factors are shown at each 0.5 lag but not at Lag = 0.

## Implications

The new app enables occupational health and safety specialists to accurately and promptly monitor workers’ conditions and the organization’s status, from a broader perspective than traditional methods. In addition, every worker can monitor their own occupational health and safety status using this approach, rather than just occupational health and safety specialists. In terms of fatigue management, repeated self-checking of fatigue will also promote better working conditions and environments. Moreover, this method can serve as an objective indicator of fatigue in the future, which is recommended for consideration in the Japanese national guidelines on medical interviews for physicians with long working hours and in any other industry where fatigue monitoring is critical. However, this tool should not be used for evaluating or ranking individual employees, as the data may reflect personal cognitive characteristics. The data should be used solely to examine intra-individual variability and inter-group differences, not for comparisons between individuals.

In the academic field, the introduction of this application will enable larger-scale longitudinal surveys without being constrained by distance, costs, operational effort, and equipment shortages. The primary application is designed for internal use by the institute and is not freely available to other researchers. Although the interface is in Japanese, anyone interested in the application can request access under certain conditions^15)^. We also offer a free public version of the application with reduced functionality. The public version can perform the PVT and Flanker task and uses a visual analog scale to measure sleepiness and fatigue. As all measured data are stored in the user’s device; the server does not collect any data, except for anonymous usage statistics, from the public version. Users can export stored results as a CSV file. The public version can be found at <https://fatiguechecker.h.jniosh.johas.go.jp>.

There are some limitations associated with implementing the new app. First, the primary challenge with remote surveys, even with the new app, is ensuring participant compliance with the study protocol and rules. Participants may face challenges managing incoming calls and notifications while using their smart devices. It is essential for researchers to conduct thorough and ongoing monitoring of the collected data. Second, although the total cost of the survey will be significantly reduced, participants will need to cover the costs of internet connection and charging their battery, which should be communicated to them before participation. Finally, some participants, particularly elderly individuals, may encounter difficulties accessing the survey window and completing tasks. We have previously addressed these points by preparing detailed manuals and conducting briefings for local personnel during workplace surveys. However, these measures also incur costs, which is a disadvantage.

We have provided here an overview of a web application for survey participation that ensures low costs, high security, and stability. The application enables the self-registration and pre-registration of participants, and can be integrated with a popular messaging app for optimal communication. Since its release, it has supported six projects involving hundreds of participants and generated nearly 25,000 records, successfully collecting high-quality data and enabling researchers to monitor real-time compliance. Additionally, cross-correlation analysis based on laboratory-measured PVT data has demonstrated high temporal synchronization with data obtained using gold-standard PVT equipment. The use of the app will significantly impact occupational health and safety monitoring, enabling accurate and prompt assessments and facilitating large-scale, longitudinal surveys in the academic field at a low cost and effort. Thus, its contribution to fatigue management can potentially improve the working environment. By facilitating repeated self-checking of fatigue, this application promotes better working conditions and environments, ultimately benefiting both workers and employers.

## Supplementary Material

Supplementary eFigure 1
