# Supplementary material for: Enhanced repeated measurement of psychological tasks and form questions via a web-based mobile app
Source: Environ Occup Health Pract. 2025 Oct 9;7(1):2025-0019. doi: 10.1539/eohp.2025-0019 (PMC12738528; doi:10.1539/eohp.2025-0019)
Supplement: Supplementary file 1 — Supplementary eFigure 1 [file eohp-7-2025-0019-s001.pdf]

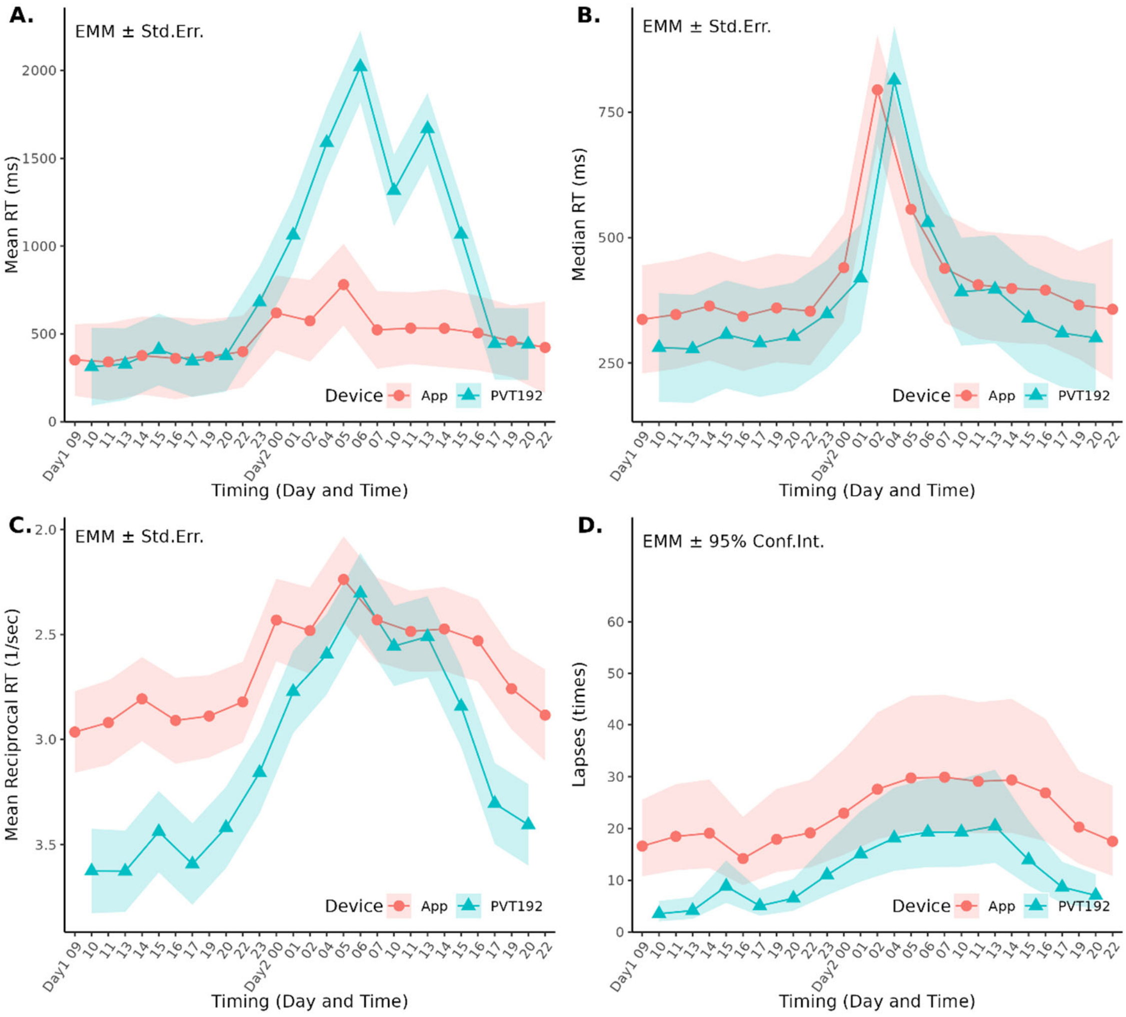

eFigure 1. Time series of the PVT measures

A: Mean Reaction Time (RT), B: Median RT, C: Mean Reciprocal RT, and D: Number of lapse trials for the app and the PVT-192 are shown in the figure. The significant raw value difference between the two devices might have originated from different user interfaces (touch screen and physical button) and task settings (eg, task duration 3 and 10 minutes for the app and the PVT-192; lapse threshold 355 ms and 500 ms, respectively).
